# Insecticidal Activity of Plant Lectins and Potential Application in Crop Protection

**DOI:** 10.3390/molecules20022014

**Published:** 2015-01-27

**Authors:** Maria Lígia R. Macedo, Caio F. R. Oliveira, Carolina T. Oliveira

**Affiliations:** 1 Department of Food Technology and Public Health, University of Mato Grosso do Sul, Campo Grande, Mato Grosso do Sul 79070-900, Brazil; E-Mails: oliveiracfr@gmail.com (C.F.R.O.); caroltuo@hotmail.com (C.T.O.); 2 Department of Biochemistry and Tissue Biology, University of Campinas, Campinas, Sao Paulo 13083-862, Brazil

**Keywords:** lectin, insect, pest control, crop protection

## Abstract

Lectins constitute a complex group of proteins found in different organisms. These proteins constitute an important field for research, as their structural diversity and affinity for several carbohydrates makes them suitable for numerous biological applications. This review addresses the classification and insecticidal activities of plant lectins, providing an overview of the applicability of these proteins in crop protection. The likely target sites in insect tissues, the mode of action of these proteins, as well as the use of lectins as biotechnological tools for pest control are also described. The use of initial bioassays employing artificial diets has led to the most recent advances in this field, such as plant breeding and the construction of fusion proteins, using lectins for targeting the delivery of toxins and to potentiate expected insecticide effects. Based on the data presented, we emphasize the contribution that plant lectins may make as tools for the development of integrated insect pest control strategies.

## 1. Introduction

More than a century has passed since the first studies regarding plant compounds with hemagglutinating activity that constitute the complex group of proteins currently known as plant lectins. A series of studies have continually implemented novel insights regarding the structure and function of this huge plant protein family. In this review, we present some of these findings and demonstrate the biotechnological potential of plant lectins for pest control, corroborating the practices of the integrated pest management (IPM).

The first description of a compound with hemagglutinating activity was reported in 1888, when Stillmark described a toxic factor extracted from castor beans (*Ricinus communis* L.) with the capability to agglutinate red blood cells from different animals [1]. He attributed both the toxicity and agglutinating activity to this factor, which was named ricin. Later, with the improvement of protein isolation techniques, the first lectin was isolated from *Canavalia ensiformis* seeds, also known as Jack beans. The protein was called concavalin A (ConA) [2] and was the first lectin isolated in crystalline form. Subsequently, the ability of this protein to agglutinate erythrocytes, yeast cells, bacteria and other compounds, such as starch granules, could be extensively examined [3]. For a long period, these proteins were denominated hemagglutinins, due to their ability to agglutinate erythrocytes and other kind of cells. However, it was later discovered that some lectins did not exhibit this agglutinating activity. The term lectin was then coined and has been used since. The word lectin originates from the Latin word *legere*, which means *select*. The current definition for plant lectins proposes the presence of at least one non-catalytic domain that binds reversibly to a specific mono- or oligosaccharide [4], and this property constitutes the basic criteria for the classification of a protein as a plant lectin, while agglutination is no longer used for such classification criteria. Furthermore, lectins can also demonstrate additional domains, beyond the sugar-binding domains that confer different biological activities [5].

## 2. Structural Characteristics and Classification of Plant Lectins

Based on their number domains and their characteristics, plant lectins can be divided into four classes. The simplest class, the merolectins, are lectins that possess a single carbohydrate-binding domain. As a result, the merolectins do not present agglutinating activity. Hololectins contain two or multivalent carbohydrates-binding sites. The third class of lectins, the chimerolectins, possess a carbohydrate-binding domain and an additional domain that confers other biological activities. Finally, the superlectins are lectins with two or multivalent carbohydrate domains that are able to recognize structurally unrelated sugars [6].

Currently, the data from genome and transcriptome studies indicate that most of the plant lectin genes have a chimeric domain architecture, reinforcing the idea of a group with bi- or multi-functional biological roles [7]. In addition, a number of lectins from rice, soybean and *Arabidopsis* contain additional domains besides the carbohydrate-binding domain, such as a Kinase domain, suggesting that complex functions for these proteins have been acquired during the evolution of plants [6].

Due to advances in plant biochemistry and molecular biology techniques, over the last two decades, the plant lectins have been classified based on their carbohydrate-binding domains. In 1998, a novel classification system was proposed, grouping the lectins into seven distinct families, considering both sequence similarities and their evolutionary relationships [6]. Since 1998, five novel lectin domains have been identified in plants. At present, plant lectins are classified into 12 different families, with distinct carbohydrate-binding domains. The families are: *Agaricus bisporus* agglutinin homologs, *Amaranthins*, class V chitinase homologs, *Euonymus europaeus* agglutinin family, *Galanthus nivalis* agglutinin family, proteins with hevein domains, jacalins, proteins with legume lectin domains, LysM domain proteins, the *Nicotiana tabacum* agglutinin family and the ricin-B family.

To date, all plant lectins belong to one of these 12 families, with the exception of an exclusively maltose-binding lectin isolated from *Dioscorea batatas* (DB3L), which could not be classified into any known plant lectin family in terms of both structure and sugar specificity. This lectin shows sequence homology to dioscorin B from *Dioscorea alata* with 90% identity and to carbonic anhydrase from *Arabidopsis thaliana* with about 45% identity [8]. How DB3L acquired its hemagglutinating activity remains unknown.

In general, the three-dimensional structure of lectins is composed of a high content of β-sheets with little contribution from α-helixes. The β-sheets are connected by loops forming antiparallel chains. The stability of dimers and tetramers is conferred by hydrophobic interactions, hydrogen bonds and salt links [9]. The lectin carbohydrate-binding sites can be grouped into three overlapping regions. The central region is constituted by a conserved core in which residues interact with metallic ions (Mn^2+^ and Ca^2+^), required for carbohydrate interactions. This core makes little contribution to the lectin’s carbohydrate specificity; however, it provides necessary binding energy. Surrounding the core, aromatic residues occupy variable positions in a horseshoe shape. This region is involved in the lectin’s monosaccharide specificity. Finally, residues with higher variability are located in the outer zone and are involved in interactions with larger oligosaccharide ligands [9,10].

## 3. Constitutively Expressed and Inducible Lectins

Why plants express lectins has intrigued researchers for a long time. Lectins have been detected in hundreds of different plant species and the first studies characterized lectins from mature seeds [9,11]. Vegetative tissues, such as bulbs, tubers, rhizomes, roots, bark, stems, fruits and leaves [5,6] are also rich sources of lectins. Furthermore, lectins possess a broad distribution in plant tissues, and the amounts accumulated in each tissue and species vary in the same proportion.

The role of lectins in plant physiology is unclear. As lectins are often abundantly concentrated (generally 0.1%–10% of the total protein) in seeds or vegetative storage tissues (e.g., bulbs, bark and rhizomes), these proteins are thought to act as storage proteins, representing a source of amino acids for rapid growth and development [6]. The constitutively expressed lectins are also referred to as such “classical lectins” [5]. Accumulating data have revealed that the majority of these lectins accumulate in vacuoles or related organelles, or are secreted into the extracellular compartment [7]. Those lectins that are particularly expressed at high concentrations are also considered to be defense-related proteins against pathogens and herbivorous insects [12,13,14]. These features indicate that plant lectins possess multifunctional roles as supported by the fact that most lectins have a preferential or even exclusive specificity for foreign over plant-specific glycans [15]. The influence of symbiosis with microorganisms has also been studied [16].

In contrast to highly-expressed lectins, another lectin group is expressed under specific conditions and at lower concentrations and are probably involved in further functions and mechanisms currently not fully understood [17]. During the last decade, this new class of plant lectins denominated the “inducible lectins” has been extensively studied. Under normal growth conditions, or in the absence of stress factors, the inducible lectins are not expressed at detectable levels. In contrast to classical lectins, most inducible lectins remain in the nucleus and the cytoplasm of the plant cell [5,18]. The first inducible lectin to be purified and characterized was found in rice seedlings and named *Oryza sativa* agglutinin (Orysata) [19]. Orysata is a mannose-specific lectin belonging to the jacalin-related family. Although Orysata was designated as a lectin only in 2000, ten years earlier Claes *et al.* [20] described Orysata as a salt-inducible protein (SalT). The salT mRNA accumulates rapidly in sheaths and roots of mature plants and seedlings upon salt or drought stress. Later, Moons *et al.* [21] showed that salT expression was also induced by abscisic and jasmonic acid.

In a recent study, Orysata was successfully expressed in transgenic tobacco and studied with respect to its insecticidal activity against three important pest insects: the beet armyworm (*Spodoptera exigua*), the green peach aphid (*Myzus persicae*) and the pea aphid (*Acyrthosiphon pisum*). The latter was fed on an artificial diet containing different concentrations of Orysata. Results demonstrated that Orysata possesses strong insecticidal activity. Larvae of *S. exigua* fed on transformed plants presented higher larval mortality, reduced weight and extension of larval development. Similar to the beet armyworm, *M. persicae* also exhibited higher mortality compared to aphids fed on the wild type plants. Moreover, a low LC_50_ value (79 µg/mL) in the pea aphid (*A. pisum*) bioassay indicates that Orysata has strong negative effects on this insect, compared with other mannose-binding lectins [12].

Studies of changes in wheat (*Triticum aestivum*) gene expression during infestation by Hessian fly (*Mayetiola destructor*) larvae have identified three lectin-like genes expression, suggesting their involvement in the defense response. These genes are designated as *Hfr* (Hessian fly responsive) and promote the expression of a high mannose N-glycan-specific jacalin-like lectin, and two other lectin-like proteins with an amaranthin domain and a chitin-binding hevein domain, named HFR1, HFR2 and HFR3, respectively [22,23,24].

Williams *et al.* [22] reported that infested wheat-resistant plants accumulate higher levels of *Hfr-1* transcripts from one to three days after egg hatching, compared with uninfested or susceptible plants. This period corresponds to the period when first instar larvae inject substances into the plant in an attempt to establish the feeding site. Interestingly, resistant wheat plants accumulate high levels of HFR1 at the larval feeding sites [22]. When infested by *M. destructor*, wheat plants also presented high expression levels of HFR2 and HFR3 [23,25].

In another approach, HFR1 and HFR3, produced as recombinant proteins, showed insecticidal activity towards another major wheat pest, the cereal aphid *Sitobion avenue*, which does not invoke gene-for-gene responses in wheat plants, such as the Hessian fly. Aphids fed on a liquid diet containing HFR1 and HFR3 showed a significant reduction in survival and growth. The authors also observed that HFR3 binds to the midgut region in cereal aphids and is stabilized against degradation by gut enzymes for up to 48 h [26].

Another inducible lectin was purified in 2002 from tobacco (*Nicotiana tabacum*) leaves, and further referred to as *N. tabacum* agglutinin or NICTABA [27]. NICTABA was one of the first nucleocytoplasmic lectins characterized in plants [18]. Its expression is induced in leaf parenchyma cells after treatment with jasmonates, known phytohormones involved in defense responses. Vandenborre *et al.* [28], reported that chewing caterpillars (*Spodoptera littoralis* and *Manduca sexta*) and cell-content-feeding spider mites (*Tetranychus urticae*) also induce NICTABA expression. On the other hand, infestation with phloem-feeding herbivores (*Myzus nicotianae* and *Trialeurodes vaporarorum*) does not result in NICTABA accumulation. These differences suggest that feeding behavior plays an important role in the regulation of NICTABA expression, since phloem feeders cause little or minor damage to plant tissues, in comparison with mites and chewing caterpillars [29].

## 4. Anti-Insect Activity and Mode of Action of Plant Lectins

Of the several biological functions of plant lectins [30], their anti-insect activities have received particular attention [17,31]. Over the last few years, the development of plants resistant to insect attacks has opened a promising field for the use of plant lectins in pest management strategies [32,33]. The use of lectins in transgenic plants has yielded positive results, especially for crops now expressing Cry toxins from *Bacillus thuringiensis* (*Bt*), which demonstrate resistance to sap-sucking insects. Furthermore, the ingestion of lectins in artificial diets or their expression in transgenic plants has been shown to reduce performance in insects belonging to different orders, including Lepidoptera, Coleoptera, and Hemiptera [31]. Although a number of studies have addressed lectin toxicity, the exact mode of action of lectins in providing resistance against insects remains unclear.

**Figure 1:**
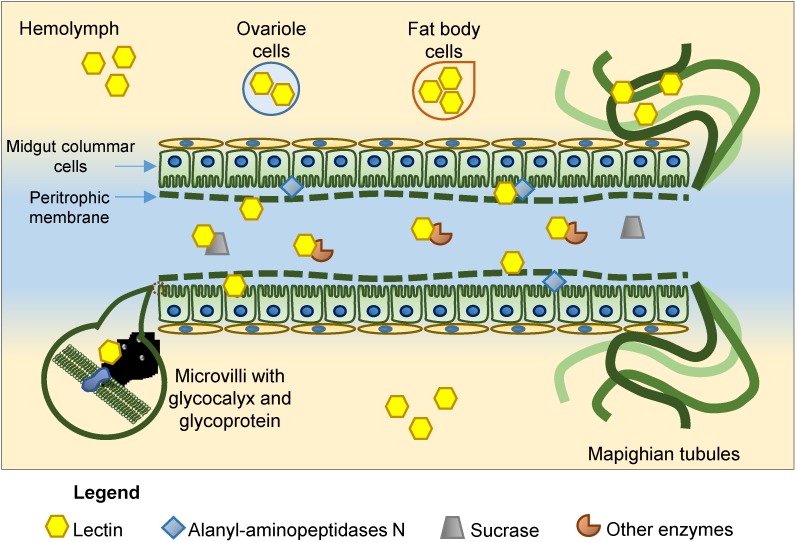
Representative scheme showing the binding sites of plant lectins that have been reported. In addition to the sites displayed in the figure, other receptors are also known: α-amylase, α- and β-glucosidases, trypsin-like enzymes, ferritin, NADH quinone oxidoreductase, vacuolar ATPase; sarcoplasmic reticulum type calcium ATPase, heat shock protein 70, β-subunit ATP synthase and Clathrin heavy chain.

The most likely mechanisms underlying the entomotoxic activity of lectins involve interactions with different glycoproteins or glycan structures in insects, which may interfere with a number of physiological processes in these organisms. Since lectins possess at least one carbohydrate-binding domain and different sugar specificities, and considering the variety of glycan structures in the bodies of insects [34,35], possible targets for lectin binding are numerous (Figure 1). Therefore, it is difficult to predict the exact mode of action of each lectin and even more difficult to understand the variability in insect toxicity upon exposure to different plant lectins.

Broadly speaking, antinutritional effects are often observed as a result of lectin ingestion and could affect several biological parameters in insects, such as larval weight, fecundity, pupation and survival. Likewise, an extension in the larval development period and adult emergence is commonly observed [36]. It is important to note that one of the principal characteristics of an insecticidal protein constitutes its resistance to proteolytic degradation in the insect gut [37]. In general, plant lectins present a high resistance to insect digestive enzymes. For example, *Moringa oleifera* lectin (cMoL) is resistant to digestion by *Anagasta kuehniella* proteases for up to 12 h [36] and *Olneya tesota* lectin (PF2) is not degraded by *Zabrotes subfasciatus* digestive enzymes within 24 h [38]. NICTABA shows an even stronger resistance and is not digested for up to three days of incubation with midgut extracts of *S. littoralis* [39].

For some time, efforts have focused on finding more efficient ways to control sap-sucking hemipteran attacks on several important crops [40]. These insects cause dramatic negative impact on plants, especially due to their extremely rapid population growth [41]. Studies of plant lectin effects on Hemipteran pests have provided interesting results regarding their mode of action. The snowdrop lectin (*Galanthus nivalis* agglutinin or GNA) has received particular attention due to its toxic effects against hemipterans and other economically important insect pests. Through immunohistochemical studies, Powell *et al.* [42] showed GNA binding to cell surface carbohydrate moieties in the midgut epithelium of brown rice plant hoppers (*Nilaparvata lugens*). Moreover, GNA caused morphological changes in the epithelium, with disruption of the microvilli brush border region. GNA has also been observed at low levels in the fat bodies, the ovarioles and throughout the hemolymph, possibly causing general systemic effects. A depression in *N. lugens* feeding, as measured by honeydew production, was also observed when insects were fed on transgenic rice plants expressing GNA (2.0% of total soluble protein). In addition, insects had a decreased survival and overall fecundity, besides presenting a retarded development [43].

Another lectin that also displays toxicity towards hemipterans is the *Allium sativum* leaf agglutinin (ASAL) [44], a mannose-binding lectin which has been engineered into a variety of transgenic plant species, such as tobacco [45] and Indian mustard (*Brassica juncea*) [46]. Fitches *et al.* [47] demonstrated that the heterodimeric and homodimeric garlic lectins, ASAI and ASAII, produced as recombinant proteins, showed insecticidal effects when fed to pea aphids (*A. pisum*). A dose-dependent effect of these lectins was observed, with survival being most reduced at the highest lectin concentration (2.0 mg/mL). Peptide fingerprinting using MALDI-TOF mass spectrometry identified two abundant membrane-associated aphid gut proteins, alanyl aminopeptidase N and sucrase, as receptors for lectin binding. Alanyl-aminopeptidase N is glycosylated and the carbohydrate side chains contain multiple mannose residues, allowing ASA binding. Furthermore, these proteins accounted for more than 15% of the total gut protein in *A. pisum*, being predominantly associated with the microvillar membrane of the insect midgut [48]. This aminopeptidase also strongly interacts with GNA and ConA. Although binding of the lectin impairs aphid development, the lectin does not affect the insect’s aminopeptidase activity, which is thought to be associated with amino acid absorption processes [48]. Several plant lectins have also been shown to interfere with other insect digestive enzyme activities, such as α-amylase [49,50,51], α- and β-glucosidases [52] and trypsin-like enzymes [49,50,53].

Other investigations have identified the binding of GNA and ASAL to important midgut receptors of *N. lugens*, such as ferritin [54] and NADH quinone oxidoreductase [55]. These two mannose-specific lectins (ASAL and GNA) were pyramided into rice lines, which proved to be more resistant to *N. lugens*, *Nephotettix virescens* and *Sogatella furcifera*, resulting in reduced insect survival, fecundity, feeding ability and delayed development [56]. Recently, Roy *et al.* [13] reported the binding of *Colocasia esculenta* tuber agglutinin (CEA) to receptor proteins, identified as vacuolar ATP synthase, sarcoplasmic reticulum type calcium ATPase from *Bemisia tabaci* and heat shock protein 70, ATP synthase β subunit, clathrin heavy chain protein from *Lipaphis erysimi*. The changes resulting from the binding of these lectins probably entail a deficient metabolic functioning, leading to growth retardation, loss of fecundity and death.

The anti-insect activity of plant lectins towards lepidopteran pests has been reported. In a screening study, Machuka *et al.* [57] showed that 16 lectins (from a total of 25 lectins among 15 plant families) had negative effects on the legume pod-borer, *Maruca vitrata*. At least one of the parameters, such as larval survival, weight, feeding ability, pupation, adult emergence and/or fecundity was affected by lectin ingestion. GNA was one of the most active lectins, exhibiting marked effects on all parameters studied.

The negative effects of GNA on other lepidopterans have also been well documented. Larvae of *Lacanobia oleracea* fed on an artificial diet and on transgenic potatoes expressing GNA presented decreased growth besides longer instar durations [58]. GNA expressed in transgenic sugarcane decreased the performance of *Eorreuma loftini*, presenting reduced larval survival, adult emergence and female fecundity, in the first generation, and reduced pupal weight in the second generation. In contrast, deleterious effects of GNA were not observed on *Diatraea saccharalis*. Moreover, in the first generation, GNA-fed larvae showed an enhanced larval growth, but this was not observed in the second generation [59]. Despite the consistent reports of adverse effects of GNA, such effects may vary with respect to concentration of the lectin and with respect to insect species.

The insecticidal properties of the *Bauhinia monandra* leaf lectin (BmoLL) against the cowpea weevil (*Callosobruchus maculatus*), the Mexican bean weevil (*Z. subfasciatus*) and the Mediterranean flour moth (*A. kuehniella*) have been investigated. For the bruchids, BmoLL exhibited a high mortality (up to 50%) in low concentrations (0.3%–0.5% *w*/*w*) in comparison with *A. kuehniella*, with no effects on survival when larvae were fed up to 1%. At this concentration, the reduction in *A. kuehniella* larval weight was 40% [51].

Sadeghi *et al.* [60] identified ferritin as a receptor for GNA in the larval midgut of *S. littoralis*, as already mentioned above in *N. lugens*. The authors postulated that part of the insecticidal activity of GNA in insects might be due to binding and interaction with certain proteins in the digestive tract, such as ferritin, in turn interfering in the insects’ iron metabolism. In another study on *S. littoralis*, Caccia *et al.* [61] showed that the lectin from *Hippeastrum* hybrid (*Amaryllis*) bulbs, named HHA, interacts with the brush border of midgut cells. FITC-HHA showed that the labeled lectin binds to the cell membrane of columnar cells and is also internalized into columnar cells. This suggests that HHA is able to cross the gut epithelial barrier and pass into the insect hemolymph. In addition, the authors showed that the FITC-HHA internalization mechanism involves clathrin-mediated endocytosis.

Differences in gene expression profiles have been reported in insects fed on a lectin-containing diet. Li *et al.* [62] reported that *Drosophila melanogaster* larvae exposed to a dietary WGA evoked differential expression of 61 transcripts; among these were several chitin-binding transcripts. The authors hypothesized that the midgut responds to the damage caused by WGA to the structure of the PM, producing more chitin for repair or replacement of PM. Other differentially-transcribed gene clusters were associated with digestive enzymes, cytoskeleton organization, detoxification reactions and energy metabolism. Such studies of gene expression analysis may contribute to the better understanding of the broad mode of interaction of plant lectins and insects.

Another possible target site for lectins in the gut region is the peritrophic membrane (PM), since this membrane is mainly composed of chitin-microfibrils and glycoproteins [63]. In contrast, hemipterans do not have a PM. In the case of lepidopterans, a lectin-containing diet can cause deformations in PM formation and disruption of the microvilli. When the European corn borer (*Ostrinia nubilalis*) was fed on a wheat germ agglutinin (WGA)-containing diet, larvae presented hypersecretion of unorganized PM in the anterior midgut as well as disintegration of microvilli, indicating the binding of WGA to the chitin microfibers and several glycosylated matrix proteins [64]. The interaction of WGA may cause voids in the PM, allowing the passage of food particles through the PM and penetration into the microvillar brush border. On the other hand, the same was not observed for *M. sexta*, which presents differences in PM formation and structure, suggesting that these characteristics affect the interaction of WGA with chitin and other components of the PM [65]. WGA also causes abnormalities in the microvilli of *Drosophila melanogaster* [62]. Another example is *Dioscorea batatas* agglutinin (DB1), classified as a mannose-binding lectin, which strongly binds to the *Helicoverpa armigera* larvae brush border and the peritrophic membrane [66].

Recently, in an interesting study, Walski *et al.* [67] evaluated the toxic effects of three lectins that were structurally related but had different molecular sizes. The lectins studied were *Sambucus nigra* agglutinin I and II (SNA-I and SNA-II) and *Rhizoctonia solani* agglutinin (RSA) (a fungus lectin), with molecule sizes of 240, 32 and 15.5 kDa, respectively. *In vitro* experiments with the *Tribolium castaneum* cell line indicated a high cytotoxicity for all three lectins, with SNA-II presenting the most toxic effect, followed by SNA-I and lastly RSA. Curiously, when the lectins were introduced into the larvae diet at 2% (*w*/*w*), RSA produced the highest toxic effect *in vivo*. Searching for the mechanisms underlying the differences in lectins toxicity, the authors found that RSA passed through the PM more efficiently than SNA-II. Moreover, both lectins were shown to be resistant to proteolysis, in contrast to SNA-I, which is thought to be degraded in the gut. SNA-II presented a partial permeability, which may be attributed to its monomeric form in solution, allowing it to cross the PM, while the oligomers with larger dimensions than the pore membrane are retained in the endoperitrophic space. These findings indicate that the ability to pass through the PM, according to the lectin’s molecular size and size of PM pores, is a key feature for lectin toxicity. It is also known that the entomotoxicity of SNA-I and SNA-II against lepidopteran and hemipteran insects is related to the induction of apoptosis via a caspase-dependent pathway in the insect gut [68,69].

## 5. Biotechnological Applications

Insects resistant against single-defense genes, such as those currently used in *Bt*-crops, are overwhelmingly reported [1,5,70]. Thus, the combination of proteins with different modes of action, as well as the correct application of IPM has the potential to improve resistance against insects over the long-term. Based on a series of reports that has shown major effects of plant lectins on insect development, the use of plant lectins in combination with other toxic proteins has been proposed in order to obtain a synergistic effect.

Among the most studied lectins purified from the snowdrop bulbs is *G. nivalis* (Amaryllidaceae). The *G. nivalis* agglutinin, or GNA, is composed of four identical 12 kDa subunits with affinity for α-1,3- or 1,6-linked d-manose residues in carbohydrates [71]. Artificial diet feeding assays have shown the insecticidal effects of GNA for several insects, including sap-sucking insects. Since the major pests for rice are Homopteran, the brown plant hopper (*N. lugens*) and green rice leafhopper (*N. virescens*), transgenic rice plants demonstrating tissue-specific expression of GNA were constructed and evaluated for control of the brown plant hopper [43]. Insects fed on transformed plants exhibited a significant decrease in survival and fecundity, which retarded their development. GNA also exhibited insecticidal effects for the glasshouse potato aphid (*Aulacothum solani*) through transformation of potato plants [72]. GNA expression level of 0.3%–0.4% provoked a significant reduction in both survival and fecundity in glasshouse bioassays.

Transgenic plants expressing GNA were also fed to the white backed plant hopper (*S. furcifera*), showing a remarkable effects on insect survival, fecundity and plant damage [73]. Western blot analysis showed that the GNA expression was 0.3% of the total soluble protein. At this level, a reduction of 90% in survival was recorded for nymphs fed on transgenic plants. Among the survivors, only 25% reached the adult stage and these showed a reduction of 90% in average fecundity. The negative effects of GNA promoted a reduction of 88% in feeding rate, in comparison with insects fed on control plants.

The insecticidal activity and involvement of *N. tabacum* agglutinin (NICTABA) in tobacco defenses have been investigated [39]. In this study, the NICTABA gene was silenced in *N. tabacum* using the RNAi technique. Moreover, the NICTABA gene was expressed in *Nicotiana attenuata*, a species that does not possess genes encoding NICTABA. *S. litorallis* larvae fed on NICTABA-silenced plants showed an increase of 70% in average weight compared with larvae fed on wild-type tobacco. When the larvae were fed with NICTABA-expressing plants, a similar reduction in *S. littoralis* larval weight was recorded (~70%). Bioassays with *M. sexta* larvae also corroborated the reduction of larval weight with the presence of NICTABA in plant tissues. With these bioassays, the authors demonstrated the involvement of NICTABA in the defense of tobacco against insect pests.

Studies evaluating the synergic effect of plant lectins and other proteins have been carried out, as well as studies to explore carbohydrate specificity in order to carry other toxic proteins to active sites. The pea lectin (P-Lec) and the cowpea trypsin inhibitor (CpTI) were expressed in tobacco plants and protection against the tobacco budworm (*Heliothis virescens*) evaluated [74]. In comparison with plants expressing only P-Lec (C^−^L^+^) or CpTI (C^+^L^−^), tobacco plants expressing both defense genes (C^+^L^+^) showed a higher mortality rate and the lowest larval weight. In addition, the leaf damage was markedly reduced, in contrast with control plants or the other treatments.

In 2003, Zhu-Salzman *et al.* [75] constructed a recombinant fusion protein from the soybean cysteine protease inhibitor N (scN) and *Griffonia simplicifolia* lectin II (rGSII). The individual proteins did not present insecticide effects on the cowpea bruchid (*C. maculatus*) development. Subsequently, they compared the insecticide activity of direct protein mixtures when used with a fusion protein, called scN-rGSII. After purification of the fusion protein, the authors demonstrated that both the inhibitory activity and carbohydrate binding were preserved. Bioassays showed that the insecticide activity of scN-rGSII is higher than that of the mixture protein at the same concentration, justifying the use of the fusion protein. At the higher concentrations evaluated (0.67% scN-GSII), the fusion protein induced 100% mortality, while feeding on the separate proteins led to a mortality of 50% on average. The authors suggested that the fusion protein could block the access of scN-degrading proteases, as described in the cowpea bruchid [76], and indicated that different proteins may possess domains with similar or different functions. Through this approach, it is possible to achieve a higher insecticidal activity and control strategy durability, compared to the use of single defense genes.

Some lectins exhibit a strong resistance to gut proteolysis, probably to maintain the protein’s carbohydrate affinity. This characteristic can be taken advantage of to explore the delivery of these proteins at other sites, such as the hemolymph. The application of lectins as such protein carriers has been explored for the delivery of a number of compounds, such as hormones and toxins. The potential of these compounds when ingested orally is quite low, since they need to reach the hemolymph to exert their insecticidal effects. Different insecticide hormones have been studied in order to become alternatives for pest management. Due to their low dose and specificity, these compounds may be able to control many physiological and metabolic aspects, such as biosynthesis and muscular controls [77,78]. The injection of *M. sexta* allatostatin (Manse-AS) in *L. oleracea* larvae was found to result in a high mortality rate (up to 80%) while oral feeding did not present any effect [79]. In order to evaluate the potential of GNA as a carrier for Manse-AS into the *L. oleraca* hemolymph by oral ingestion, Fitches and co-workers [80] constructed a GNA/Manse-AS fusion protein. Immunoreactivity assays showed high activity for GNA/Manse-AS fed larvae and size exclusion chromatography demonstrated that this activity was due to a fraction with a molecular weight that was similar to that of the intact fusion protein. In other words, the fusion protein is able to cross the gut epithelium and reach the hemolymph without signals of degradation. This promising result highlights the potential for the use of plant lectins as protein carriers.

Another fusion protein combination was investigated using the biological properties of GNA for the delivery of ButaIT, a toxin from the red scorpion *Mesobuthus tamulus* [81]. The feeding bioassays with ButaIT/GNA were carried out with *S. littoralis*, *Musca domestica* and *T. castaneum*. For *S. littoralis*, ButaIT/GNA-fed larvae showed a mortality of 30% within 24 h. At the end of the experiments, the most interesting result was the marked low weight of the ButaIT/GNA-fed larvae, which was 88% lower in comparison with control-fed larvae. For assays with *M. domestica*, ButaIT/GNA provoked a mortality rate of 35%, 66% and 75% after 24, 48 and 72 h, respectively. *T. castaneum* larvae fed with ButaIT/GNA showed a decrease in average weight of 90% at the end of the experiments and presented a mortality of 50%. Thus, authors demonstrated the application of GNA as a carrier protein for facilitating the delivery of neurotoxins into the circulatory system. Similar approaches are attractive, since the insect hormones and neurotoxins do not offer risks for higher animals and are effective and potentially applicable for pest management.

Another neurotoxin highly insecticidal to a variety of insect pests, the spider-venom peptide ω-hexatoxin-Hv1a (Hv1a), has a limited oral toxicity. Since GNA has been proved to be resistant to proteolytic activity and when ingested it is transported into the hemolymph, Fitches *et al.* [82], investigated the ability of GNA as a carrier for Hv1a. The authors showed that the Hv1a/GNA fusion protein had significant oral activity against the lepidopteran *Mamestra brassicae*. Even more interestingly, the authors presented evidence for binding of orally delivered GNA to the lepidopteran central nervous system, which may facilitate the toxin´s action. Recently, another study by Nakasu *et al.* [83], demonstrated that Hv1a/GNA was not toxic to the non-target species, *Apis mellifera*. Although the larvae internalized the Hv1a/GNA toxin, the survival rate was only slightly affected (LD50 > 100 µg bee-1). Likewise, no adverse effects of the fusion protein on honeybee learning and memory were observed.

Through a similar approach, Yang *et al.* [84], demonstrated the efficiency of GNA as a carrier fused to another spider venom peptide, δ-amaurobitoxin-Pl1a. When third instar larvae of *M. brassicae* were fed on the fusion protein (30 µg), 100% larval mortality was noticed within six days. The P11a/GNA also caused increased mortality when injected in housefly (*Musca domestica*). *A. pisum* nymphs fed on a Pl1a/GNA-containing diet (1 mg/mL) presented 100% mortality after three days of feeding. The Pl1a/GNA labeled by conjugation with fluorescein demonstrated binding to the aphid gut and persistence in the midgut region even after 48 h.

Recently a new synthetic gene was constructed, comprising a combination of *Bt* toxin Cry1Ac and the *Allium sativum* agglutinin (ASAL) [85]. In bioassays, Cry-ASAL showed enhanced insecticidal activity for *Pectinophora gossypiella* and *H. armigera*, being 8- and 30-fold more toxic for these insects when compared with Cry1Ac feeding. The increase of the entomotoxic effect observed for Cry-ASAL was attributed to its binding to additional receptors in the midgut cells, mediated by the carbohydrate-binding sites of ASAL.

## 6. Tri-Trophic Interaction and Safety Assessment of Lectin-Expressing Plants

Many research groups are seeking different plant genes with insecticidal effects for biotechnological purposes. However, the impact of the insertion of a new gene on natural pollinators and the development of beneficial predators has not been investigated with the same frequency. The potential to use lectin-mediated tri-trophic effects for pest control has been studied. However, few results are available and these vary according to the lectin, the target pest and it respective natural predator.

The tri-trophic effect of GNA, when expressed in potato plants, on the two-spot ladybirds (*Adalia bipunctata*), the natural predator of peach-potato aphids (*M. persicae*), has been investigated [86]. The ladybirds were fed with aphids previously fed on three different diets; on control potato plants, on GNA-expressing transgenic potatoes and *Vicia faba* bean plants, considered as optimal for aphid development. Subsequently, a series of physiological parameters were analyzed. No difference in aphid consumption (in terms of weight eaten) by ladybirds was observed between the treatments. However, both egg laying and fertility were significantly reduced in the ladybird group fed with GNA-aphids. Furthermore, the ladybirds fed with GNA-aphids showed a 50% reduction in longevity. The authors suggested that the accumulation of GNA in the insect gut might exert a direct effect on the ladybirds’ performance. Interestingly, when the ladybirds were fed with aphids from control plants, the parameters of weight and longevity improved. The authors highlighted the importance of “refuge” areas, at which non-transgenic plants should be planted in a specific arrangement. The adoption of this measure would have two positive impacts: first, it would decrease the selection pressure on the target pest, hindering the breakdown of the plants’ genetic resistance. Secondly, the availability of unaffected aphids would corroborate the ladybirds’ feeding range.

The effect of insecticide proteins on development of the green lacewing, *Chysoperda carnea* (Neuroptera: Chrysopidae), an insect used in the biological control aphids, has also been studied [87]. The feeding of *C. carnea* larvae with GNA exerted negative effects on larval development and survival. Neither the protease inhibitor SBTI nor the *Bt* toxins, Cry1Ab and Cry1Ac, exerted negative effects on *C. carnea* larval development, while the main effect observed in GNA-treated larvae is reduced fertility in adults insects [88].

Although the predators’ performance was affected by the presence of lectin-expressing transgenic plants, similar effects are also observed when the defenses of non-transformed plants are triggered. For example, a seminal work evaluated the effect of tomato defenses elicited by the exogenous application of jasmonic acid (JA) on the performance of both herbivores and parasitoids [89]. The treatment of tomato plants with JA or the insect herbivory stimulates the octadecanoid pathway, culminating in the production of antinutritive compounds such as protease inhibitors and oxidative enzymes [89,90]. A direct relationship has been demonstrated between the induction of plants with JA and increases in parasitism of caterpillar pests. The models used in this study were the endoparasitic wasp *Hyposoter exiguae* and the beet armyworm, *S. exigua*. The JA-treated tomato plants demonstrated a two-fold increase in the number of wasps, in relation to the control plants. The *S. exigua* larvae fed on JA-treated plants demonstrated a reduced growth rate, compared with control larvae, and were parasitized earlier. However, the parasitoids of these larvae also showed impairments in development, such as an extension in larval development and decreased pupal weight.

The impact of transgenic plants on arthropod enemies has been critically reviewed and some provocative results have been obtained [91]. There are some discrepancies in the tests that evaluate the effects of such plants on some insect orders. Additionally, most experimental conditions did not mimic ecological conditions, since on an *ad libitum* diet, constant temperatures, single prey type and no combination of stress factors were used. Finally, small sample sizes were used in these assays, and the variability observed and the statistical analysis methods employed could endanger the conclusions regarding these tests. These findings suggest that current tests should be improved in order to increase the capability of predictions of the potential impact of transgenic crops in the environment.

Another series of tests that has received attention evaluated the biosafety of transgenic plants as a health risk for consumers before marketing. Tests with lectin-expressing plants have been carried out in laboratory animal studies in order to analyze their safety [92]. In these assays, a detailed analysis of compositional data from transgenic and parental plants is made, followed by a clinical study in which Wistar rats are fed during 90 days. In an early study, a rice variety expressing GNA was evaluated for safety [93]. Rats were fed on a diet containing 60% raw rice for 90 days. No adverse effects were reported with regard to clinical, biological, immunological, microbiological and pathological parameters. In a similar study, the immunomodulating effect of PHA-E lectin, expressed in transgenic rice, was compared with rice expressing the *Cry1Ab* toxin from *Bacillus thuringiensis* (*Bt*), a toxin already commercialized in different crops [94]. PHA-E-fed rats presented immunomodulatory effects. An increase in mesenteric lymph node weight was observed, suggesting local effects in the intestine. No immunotoxicological effects were observed for *Bt*-fed rats. The authors concluded the study by reinforcing the importance of evaluating the sensitization of consumers prior to the introduction of new genes to the world market. Complementary studies evaluating the effect of cooked food, such as rice or other seeds, were not made and it is possible that the cooking process may affect the activity of expressed proteins and should be investigated for a global view regarding the production of a new transgenic crop. To date, lectin-expressing crops have been constructed for research proposes only, with no variety being currently commercially available.

## 7. Conclusions and Future Directions

Over the next few years, an increase in world food production is expected. The implementation of agricultural machinery and food technology plays a prominent role in this increase, as well as the amplification of planted land. With the wide food supply, insect attacks will probably increase in the same proportion. Approaches to counter these pests are under study and will be available soon. The use of genes conferring partial resistance against insect pests could provide a more sustainable control strategy [95,96], since both selection pressure on insect resistance and the economic damage in a crop are kept at a low level [97]. Thus, the use of genes related to plant defenses, such as lectin genes, is encouraged. However, a series of factors must be carefully studied, such as, initially, the use of tissue-specific promoters for the insecticide genes, the evaluation of plants in green houses and field trials, tri-trophic effect studies, and the adoption of refuge areas in crops. Secondly, the expression of the insecticide proteins in different tissues, especially those for consumption, the manner of preparation of transgenic food (whether the food is cooked or not), and food safety should be evaluated.

As presented in this review, studies with plant lectins are advanced and, based on these findings, lectin-expressing crops should soon be commercially available. It is important to highlight that the use of insecticide genes is only one of the existing options available for pest control and this technique should be integrated with other practices, such as biological control. The potential of the incorporation of synthetic genes containing lectin domains in plants should be further investigated, since available data are promising. As such, a combination of techniques have the potential to provide alternatives for providing an adequate amount of food to the growing world population.
